# Genome-wide analysis of alternative splicing during human heart development

**DOI:** 10.1038/srep35520

**Published:** 2016-10-18

**Authors:** He Wang, Yanmei Chen, Xinzhong Li, Guojun Chen, Lintao Zhong, Gangbing Chen, Yulin Liao, Wangjun Liao, Jianping Bin

**Affiliations:** 1 Department of Cardiology, State Key Laboratory of Organ Failure Research, Nanfang Hospital, Southern Medical University, Guangzhou 510515, China; 2 Department of Cardiology, Second Affiliated Hospital of Nanchang University, Jiangxi 330006, China; 3 Department of Oncology, Nanfang Hospital, Southern Medical University, Guangzhou 510515, China

## Abstract

Alternative splicing (AS) drives determinative changes during mouse heart development. Recent high-throughput technological advancements have facilitated genome-wide AS, while its analysis in human foetal heart transition to the adult stage has not been reported. Here, we present a high-resolution global analysis of AS transitions between human foetal and adult hearts. RNA-sequencing data showed extensive AS transitions occurred between human foetal and adult hearts, and AS events occurred more frequently in protein-coding genes than in long non-coding RNA (lncRNA). A significant difference of AS patterns was found between foetal and adult hearts. The predicted difference in AS events was further confirmed using quantitative reverse transcription-polymerase chain reaction analysis of human heart samples. Functional foetal-specific AS event analysis showed enrichment associated with cell proliferation-related pathways including cell cycle, whereas adult-specific AS events were associated with protein synthesis. Furthermore, 42.6% of foetal-specific AS events showed significant changes in gene expression levels between foetal and adult hearts. Genes exhibiting both foetal-specific AS and differential expression were highly enriched in cell cycle-associated functions. In conclusion, we provided a genome-wide profiling of AS transitions between foetal and adult hearts and proposed that AS transitions and deferential gene expression may play determinative roles in human heart development.

The heart is the first organ to form and function in the vertebrate embryos^1^. The foetal heart exhibits robust proliferation while the adult heart has limited proliferative potential^2^,^3^. Soon after birth, the cardiomyocytes lose their ability to proliferate, and their subsequent growth occurs predominantly by the enlargement of pre-existing cardiomyocytes^4^,^5^. These transitions occur by transcriptional and post-transcriptional mechanisms including coordinated networks of alternative splicing (AS)^5^,^6^,^7^. Therefore, to elucidate the mechanisms of heart development and subsequently design feasible strategies to restore proliferative competence to injured heart tissues, it is necessary to understand AS transitions.

AS, a regulated process in which the same precursor mRNA (pre-mRNA) generates two or more mRNAs using different splice sites, is widespread in the genomes of humans and other species^8^,^9^,^10^. It is estimated that over 90% of human genes undergo AS, as determined by analysing the mapping of sequence reads to exon-exon junctions, with striking variation across tissue types and developmental stages^9^,^11^. The prevalence of AS has raised questions about its biological importance, and researchers are currently investigating and attempting to identify functional splicing events. In addition, recent advances in high-throughput technologies have facilitated the identification of genome-wide AS^12^. A number of genome-wide studies have found that genes exhibiting AS transitions are enriched for specific functions in different tissues.

Recently, a large-scale study revealed that AS transitions occur during late embryonic and postnatal mouse heart development, and demonstrated that protein isoform switches are important regulatory components of postnatal development^13^. In addition, alternative splicing has been shown to have central roles in cell cycle control, apoptosis, and cell fate decisions^14^,^15^,^16^. Therefore, we hypothesised that AS transitions might occur during human foetal-to-adult heart development, and these transitions may have functional importance in human heart development. However, no reports have described a genome-wide analysis of AS transitions during human heart development.

In the current study, we comparatively analysed AS and gene expression transitions of the foetal and adult heart using eight publically available RNA-sequencing datasets. In addition, we determined whether the genes exhibiting AS transitions were enriched for specific functions. Furthermore, we analysed the correlation of AS transitions and differentially expressed genes between foetal and adult hearts.

## Results

### Differential AS transitions in human foetal and adult hearts

**Figure 1:**
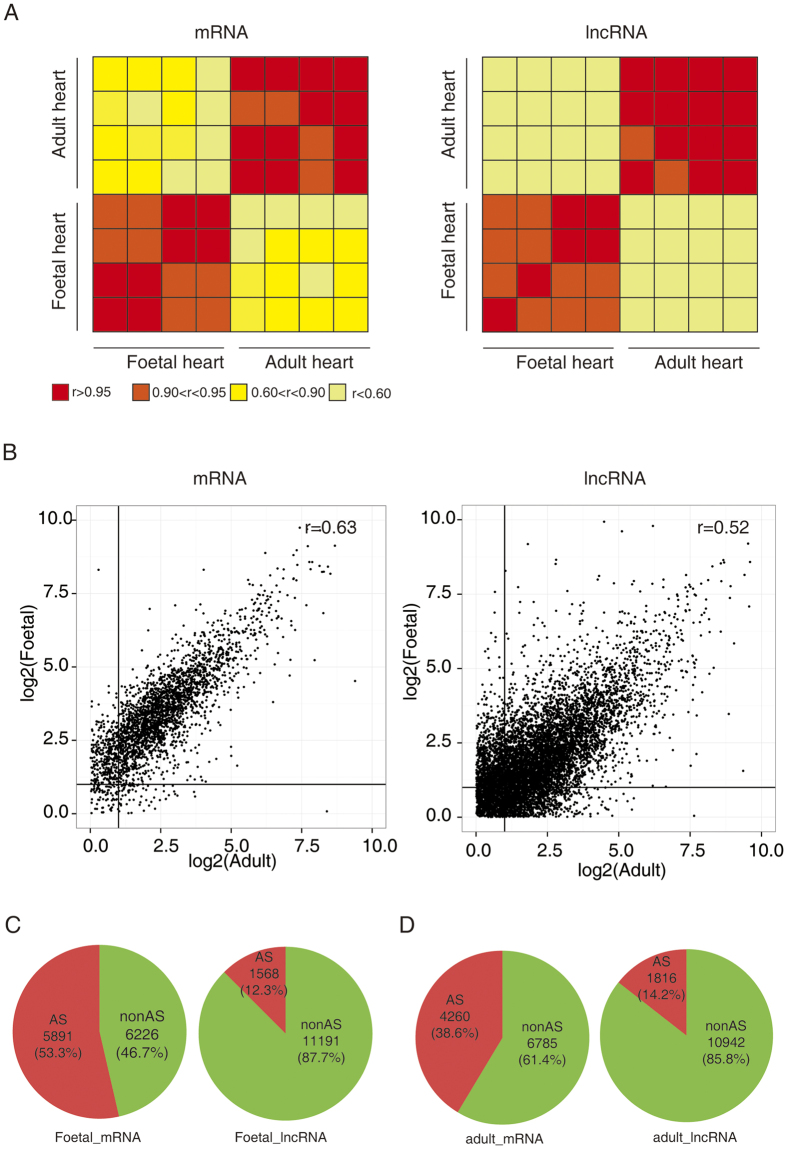
Alternative splicing (AS) transitions occurred between human foetal and adult hearts. (**A**) Heat map of mRNA and long non-coding (lncRNA) genes expression profiles of foetal and adult hearts based on correlograms visualizing correlation. (**B**) Correlation between gene transcript levels detected in foetal and adult hearts. Pearson correlation coefficients (r) are given. (**C**) Number and percentage of AS events in foetal heart. (**D**) Number and percentage of AS events in adult heart.

To identify the gene expression and AS patterns of human foetal and adult hearts, we used RNA-sequencing technology using an Illumina HiSeq 2000 instrument to analyse previously published datasets. We obtained more than 96 million reads per sample, and over 60% were uniquely aligned to the human genome (Table 1). Globally, both mRNA and lncRNA are highly concordant within groups (both r > 0.90, Fig. 1A). In contrast, there was large difference in both mRNA and lncRNA levels between foetal and adult (both r < 0.63, Fig. 1A,B).

We detected 7459 AS genes in the foetal heart, consisting of 5891 (53.3%) protein-coding genes and 1568 (12.3%) lncRNAs, respectively, that underwent AS events (Fig. 1C). In the adult heart, 6076 AS genes were identified, consisting of 4260 (38.6%) protein-coding genes and 1,816 (14.2%) lncRNAs, respectively, that underwent AS events (Fig. 1D). The AS events occurred more frequently in protein-coding genes than in lncRNAs (*P* = 0.014).

### Comparison of AS patterns in foetal and adult hearts

**Figure 2:**
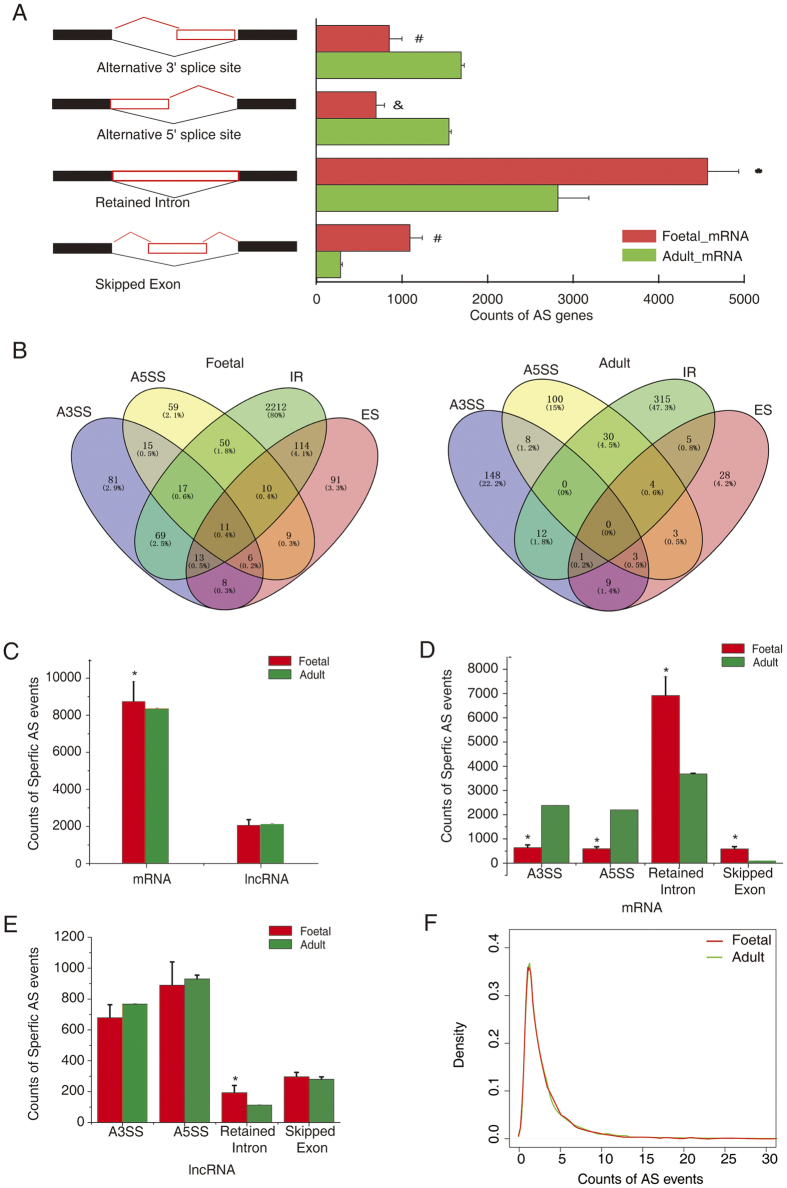
Comparison of alternative splicing (AS) patterns in foetal and adult hearts. (**A**) Difference in AS patterns between human foetal and adult hearts. (**B**) Overlap of four types of AS genes illustrated in four-way Venn diagrams of human foetal and adult hearts. (**C**) Number of specific mRNA and long non-coding RNA (lncRNA) AS events in human foetal and adult hearts. (**D**) Number of differential patterns of specific mRNA AS events in human foetal and adult hearts. (**E**) Number of differential patterns of specific lncRNA AS events in human foetal and adult hearts. (**F**) AS events per protein-coding gene. The x-axis represents number of events for each gene.A5SS, alternative 5′ splice site; A3SS, alternative 3′ splice site; AS, alternative splicing; IR, intron retention; and ES, exon skipping. *P < 0.05 versus Adult; ^#^*P* < 0.01 versus Adult, and ^&^*P* < 0.001 versus Adult.

Previous studies have revealed that alternative 3′ and 5′ splice sites, exon skipping, and intron retention account for the vast majority of AS events^17^. Therefore, we investigated these four major types of AS in both foetal and adult hearts. As shown in Fig. 2A, among the four major types of AS, intron retention (4,628 genes) was the most frequent in both foetal and adult hearts, followed by transcripts with exon skipping in the foetal heart and transcripts with alternative 3′ splice sites in the adult heart. In contrast, exon skipping was the least common AS pattern in the adult heart. We found that intron retention and exon skipping occurred more frequently in foetal hearts than they did in adult hearts (*P* = 0.015 and 0.0015, respectively). However, alternative 3′ and alternative 5′ splice sites were significantly decreased in the foetal hearts compared with those in the adult hearts (*P* = 0.0014 and 0.00016, respectively). Interestingly, the four-way Venn diagrams (Fig. 2B) illustrate a subset of overlapping genes between the four types of AS in both foetal and adult hearts. Furthermore, 11 (0.4%) and no (0%) genes exhibited all four types of AS in the foetal and adult hearts, respectively (Fig. 2B). In addition, the number of genes that underwent at least two types of AS in foetal hearts was higher than that in adult hearts (*P* = 0.014).

To identify the specific AS events in foetal and adult hearts, we compared their global splicing patterns. For all AS features identified in the heart samples, only events occurring in one group (foetal or adult) were defined as specific alternative splicing events. Using these criteria, 21232 specific AS events were detected (Fig. 2C). In general, the specific AS events were more frequent in protein-coding genes than in lncRNAs (P = 0.009, Fig. 2C). The number of the four types of specific AS genes between foetal and adult hearts was different in mRNA but not in lncRNAs (Fig. 2DE, respectively). Interestingly, among the four types of AS events, intron retention was the most frequent pattern in protein-coding genes but the least common pattern in lncRNAs (Fig. 2D,E). Although the numbers of genes that underwent AS were different between foetal and adult hearts, the average numbers of splice variants per gene were similar between them (Fig. 2F). In addition, we found that most of the protein-coding genes (98%) underwent five or fewer AS events.

### Differential AS patterns between foetal and adult hearts

**Figure 3:**
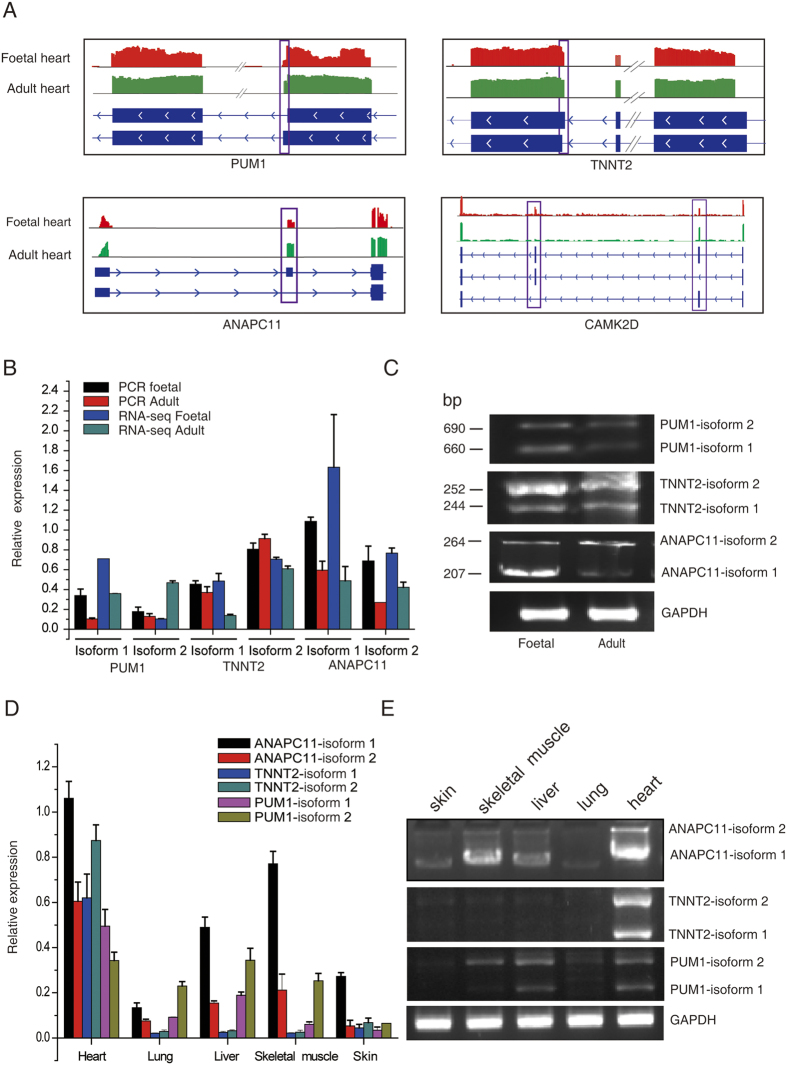
Validation of alternative splicing (AS) events using quantitative reverse transcription-polymerase chain reaction (qRT-PCR). (**A**) RNA sequencing read density plot and RT-PCR assays of AS between foetal and adult hearts. (**B**) Validation of AS events by quantitative reverse transcription-PCR. (**C**) Representative gel images illustrating the results of RT-PCR analysis in A. (**D**) RT-PCR of foetal-specific AS events of five tissues (the heart, liver, lung, skin, and skeletal muscle). (**E**) Representative gel images illustrating the results of RT-PCR analysis in D. Bars indicate standard deviation (SD). A5SS, alternative 5′ splice site; A3SS, alternative 3′ splice site; AS, alternative splicing; IR, intron retention; ES, exon skipping; PUM1, pumilio RNA-binding family member 1; TNNT2, troponin T type 2; CAMK2D, calcium/calmodulin-dependent protein kinase II delta; ANAPC11, anaphase promoting complex subunit 11; RT-PCR, reverse transcription polymerase chain reaction.

Notably, we found that several cell cycle-related genes were differentially alternatively spliced in the foetal and adult hearts (Fig. 3 and Table S1). For example, the pumilio RNA-binding family member 1 (*PUM1*), which is important for cell proliferation by repressing the E2F transcription factor 3 translation^18^, was alternatively spliced at different alternative 3′ splice sites in the foetal and adult hearts (Fig. 3A). Another cell cycle-related gene, troponin T type 2 (*TNNT2*), involved in heart development^19^ was alternatively spliced at different alternative 5′ splice sites in the foetal and adult hearts (Fig. 3A). Additionally, the AS exon skipping patterns were found to be differentially expressed between the foetal and adult hearts. As illustrated in Fig. 3A, pronounced differences in calcium/calmodulin-dependent protein kinase 2D (*CAMK2D*) and anaphase promoting complex subunit 11 (*ANAPC11*) were observed between foetal and adult hearts in the read coverage of the exons. *CAMK2D* has been shown to regulate proliferation in C2C12 cells via the Wnt5a/CaMKII pathway, and *ANAPC11* controls cell cycle regulators in numerous cells via ubiquitin chain formation and subsequent proteasomal degradation^20^,^21^.

We further performed quantitative reverse transcription-polymerase chain reaction (qRT-PCR) analysis to validate the differential splicing events of *PUM1, TNNT2* and *ANAPC11* in the human heart samples. The expression levels of both isoforms detected using qRT-PCR were similar to the results of the RNA-sequencing (Fig. 3B,C). In addition, AS events were detected in different tissues in the neonatal rats. We found that the *ANAPC11*and *PUM1*, especially isoforms 1, were highly expressed in the heart, skeletal muscle and liver, while they were lowly expressed in lung and skin (Fig. 3D,E). The TNNT2 gene was predominantly expressed in the heart, and the isoform 2 was the predominant isoform in the heart (Fig. 3D,E).

Collectively, these results suggest there are significant changes in cell cycle-related genes in AS events at different stages of human heart development.

### Foetal- and adult-specific AS events were enriched in specific functions

**Figure 4:**
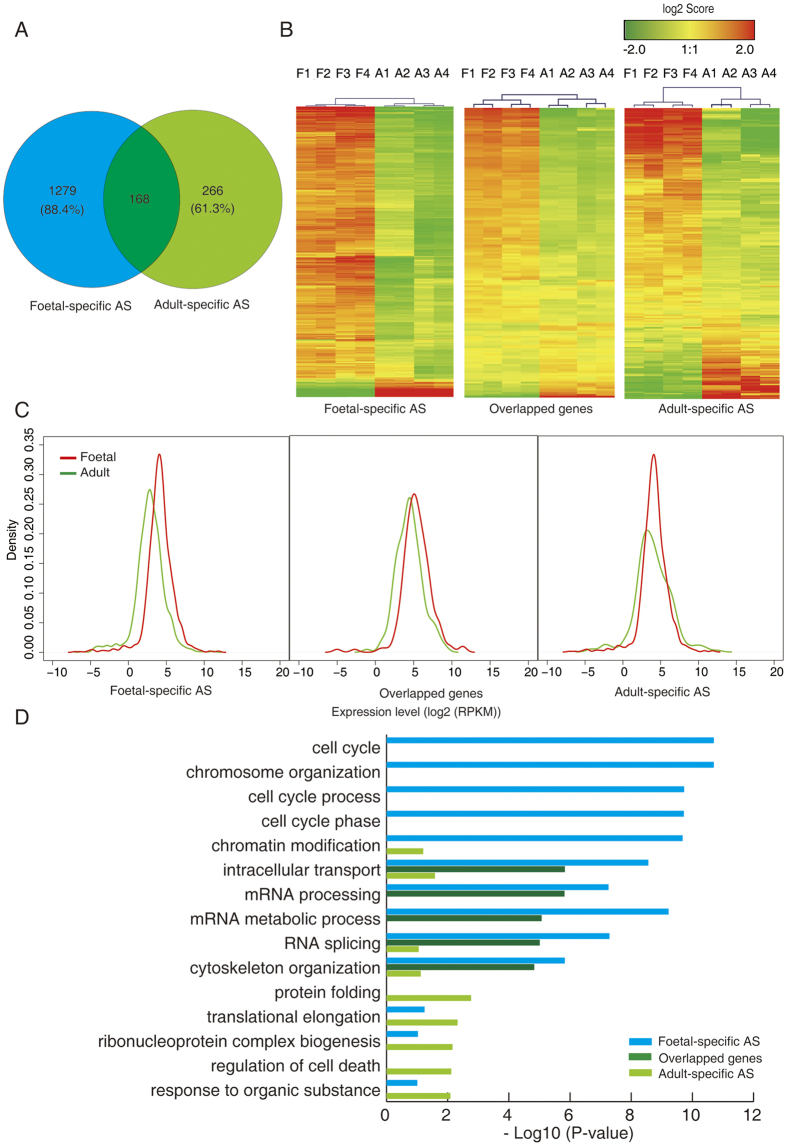
Functional categorization (biological processes) of foetal and adult-specific alternative splicing (AS) genes in human foetal and adult hearts. (**A**) Numbers and overlap of genes with foetal- and adult-specific AS events. (**B**) Heat maps showing hierarchical clustering of specific AS genes displayed in A. (**C**) Kernel density plot of transcript abundance of specific AS genes displayed in A. (**D**) Functional categories (biological process) of genes with specific AS events in human foetal and adult hearts. Foetal-specific AS, gene with specific AS events in foetal hearts; overlapped genes, overlapped genes of foetal- and adult-specific AS; adult specific AS, gene with specific AS events in adult hearts.

As illustrated in Fig. 4A, 1447 and 434 AS were identified as foetal- and adult-specific, respectively. A subset of 168 genes was found to overlap between the foetal and adult hearts (Fig. 4A). Hierarchical clustering analysis revealed that the expression of specific AS genes differed dramatically between the foetal and adult hearts (Fig. 4B). Furthermore, the Kernel density plots illustrated that the expression of foetal-specific AS products in the foetal heart was higher than in the adult hearts, while the expression of adult-specific AS genes was comparable in the foetal and adult hearts (Fig. 4C). However, no difference in expression level was found in the overlapping genes in the foetal and adult hearts (Fig. 4C).

To investigate whether the observed specific AS genes in the foetal and adult hearts exhibited specific functions, we used gene ontology (GO) category analysis and found that AS genes were enriched in the category of ‘biological processes’. Regeneration-specific categories such as the ‘cell cycle’, ‘chromosome organisation’, and ‘cell cycle process’ were found to be the most represented categories of AS genes expressed only in the foetal hearts (Fig. 4D). In contrast, translation-specific categories such as the ‘protein folding’, ‘translational elongation’, and ‘ribonucleoprotein complex biogenesis’ were found to be the most represented categories of AS genes expressed only in the adult heart (Fig. 4D). The overlapped genes in the foetal and adult hearts were mainly enriched in the category of ‘intracellular transport’, ‘mRNA metabolic process’, and ‘mRNA processing’. The analysis of Kyoto Encyclopaedia of Genes and Genomes (KEGG) pathways further demonstrated that only foetal-specific AS events were enriched in cell cycle-related functions (Supplementary Material Figure S1A).

### Specific AS and differentially expressed genes (DEGs)

**Figure 5:**
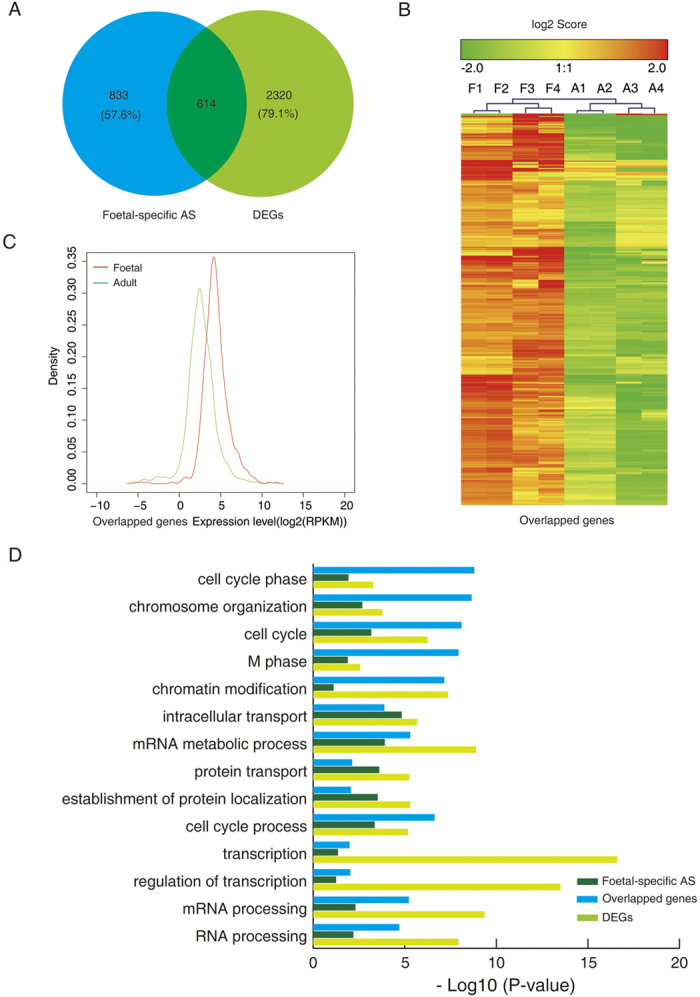
Functional categorisation (biological process) of foetal-specific alternative splicing (AS) and differentially expressed genes (DEGs). (**A**) Genes undergoing foetal-specific AS intersected with DEGs. (**B**) Heat maps showing hierarchical clustering of overlapped genes of foetal-specific AS and DEGs displayed in A. (**C**) Kernel density plot illustrating overlapped genes of foetal-specific AS and DEGs displayed in A. (**D**) Functional categories (biological process) of foetal-specific AS, overlapped genes, and DEGs displayed in A. DEGs, differentially expressed genes between foetal and adult hearts; foetal specific AS, gene with specific AS events in foetal hearts; overlapped genes, overlapped genes of foetal specific AS and DEGs.

To explore whether the DEGs and AS acted cooperatively or independently to regulate human foetal heart development, both AS and gene expression levels in foetal-to-adult transitions were simultaneously analysed. The genes with a false discovery rate [FDR] <0.05 and fold change >2 between the control foetal and adult hearts were defined as DEGs, and a total of 2,934 DEGs were identified. It is noteworthy that two important isoforms of cardiac ventricular myosin heavy chain (MHC), α-MHC (high ATPase) and β-MHC (low ATPase), were found to be differentially expressed during human heart development (Supplementary Material Figure S1B). A subset of 614 foetal-specific AS events was also differentially expressed between the foetal and adult hearts (Fig. 5A). Interestingly, the hierarchical clustering analysis of the 614 overlapped genes revealed they were all upregulated in the foetal hearts compared with those in the adult hearts (Fig. 5B). The Kernel density plot further demonstrated that the expression of these overlapped genes was higher in the foetal hearts than in the adult hearts (*P* = 1.6 ∗ 10^−5^, Fig. 5C). The GO analysis of these overlapped genes revealed that they were highly enriched in cell cycle-related functional categories (Fig. 5D). Further, KEGG pathway analysis confirmed that these overlapped genes were mainly enriched in cell proliferation-associated pathways (Supplementary Material Figure S2A). These results suggest that the overlapped genes exhibited both AS events and differential expression were critical in the regulation of human heart development.

**Figure 6:**
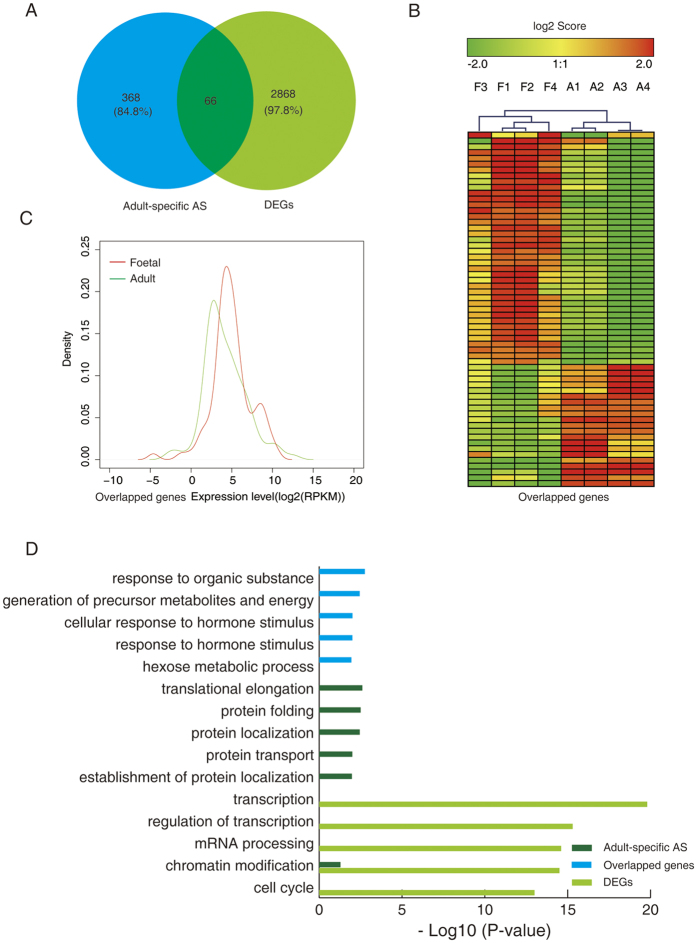
Functional categorisation (biological processes) of adult-specific alternatively spliced (AS) and differentially expressed genes (DEGs). (**A**) Genes undergoing adult-specific AS were intersected with DEGs. (**B**) Heat maps showing hierarchical clustering of overlapped genes of adult-specific AS and DEGs. (**C**) Kernel density plot illustrates transcript abundance of overlapped genes of adult-specific AS and DEGs. (**D**) Functional categories (biological process) of adult-specific AS, overlapped genes, and DEGs displayed in A. DEGs, differentially expressed genes between foetal and adult hearts; adult-specific AS, gene with specific AS events in foetal hearts; overlapped genes, overlapped genes of adult-specific AS and DEGs.

As illustrated in Fig. 6A, only 66 adult-specific AS genes also exhibited differential expression between the foetal and adult hearts. The hierarchical clustering analysis of the overlapped genes revealed that the number of upregulated and downregulated genes was similar in the foetal heart compared with that in the adult hearts (Fig. 6B). Furthermore, the expression levels of the overlapped genes found using the Kernel density plot showed no significant difference between foetal and adult hearts (*P* = 0.28, Fig. 6C). The GO analysis revealed that these genes were enriched in translation-related functional categories (Fig. 6D). In addition, the KEGG pathway analysis showed that these overlapped genes tended to be enriched in protein synthesis- and energy metabolism-related pathways (Supplementary Material Figure S2B). Notably, the over represented functional categories of foetal- or adult-specific AS differed largely from those of the DEGs (Fig. 6D,E), indicating separate regulation of AS events and DEGs during human heart development.

### Differences in functional categories between AS and non-AS genes

**Figure 7:**
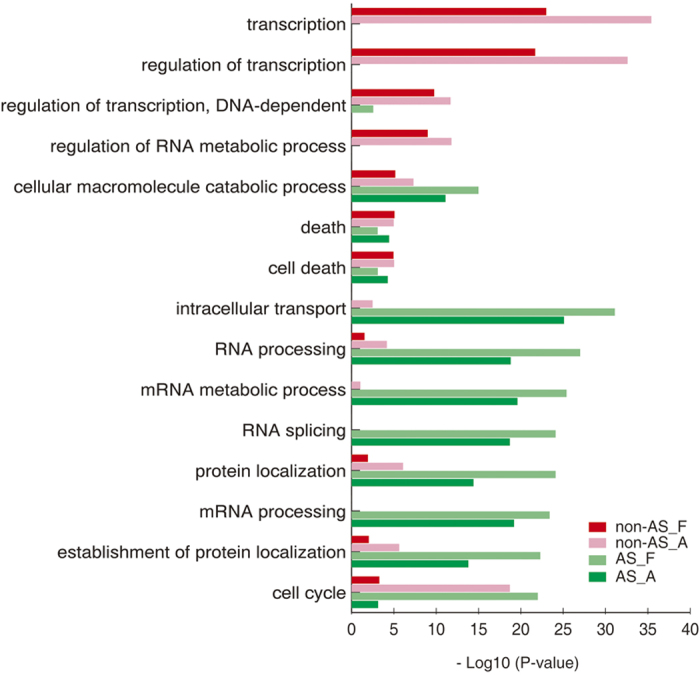
Differences in functional categories between alternatively spliced (AS) and non-AS genes. Foetal non-AS genes, genes without AS events in foetal hearts; foetal AS genes, genes with AS in foetal hearts; adult non-AS genes, genes without AS in adult hearts; adult AS genes, genes with AS in adult hearts.

We further compared the functional categories of AS and non-AS genes, and Fig. 6 shows there were pronounced differences in functional categories between them. The top three overrepresented GO categories were ‘intracellular transport’, ‘RNA processing’, and ‘mRNA metabolic process’ for AS genes, and ‘transcription’, ‘regulation of transcription’, and ‘regulation of transcription, DNA-dependent’ for the non-AS genes (Fig. 7). Interestingly, the overrepresented GO categories of foetal AS genes were comparable to those of the adult AS genes, except in the ‘cell cycle’ categories (Fig. 7). This observation reconfirmed the supposition that AS transitions play determinative roles in human heart development.

## Discussion

In this study, we presented for the first time, a high-resolution global analysis of AS transitions during human heart development. The RNA-sequencing data showed that AS patterns differed considerably between foetal and adult hearts, and the difference was mainly observed in protein-coding genes rather than in lnRNAs. The functional analysis of foetal-specific AS events showed enrichment associated with cell proliferation-related pathways, whereas the adult-specific AS events were associated with protein synthesis. In addition, genes that exhibited both foetal-specific AS and differential expression were highly enriched in cell cycle-associated functions. Therefore, we proposed that AS transitions and altered gene expression levels might play a determinative role in human heart development.

Consistent with a previous study showing the prevalence of AS in humans, our data revealed that approximately 38.6%–53.3% of the AS events occurred in the human foetal and adult hearts. The widespread occurrence of AS has been reported to increase transcriptome complexity and proteomic diversity^22^. It has been reported that the proportion of genes that produce alternative isoforms vary between tissues. Although the overall AS patterns varied across species, tissue types, and developmental stages, the intron retention has been reported to be the most common event in numerous species, including humans, plants, and mice^17^,^23^,^24^. In this study, we confirmed that the intron retention was the most common splicing type for mRNA in the human heart. The high frequency of intron retention in the human transcriptome was associated with biological functions. Previous studies have shown that intron retention is developmentally regulated and specific to the developmental phases^25^,^26^. Our data revealed that intron retention occurred more frequently in the foetal hearts than it did in the adult hearts, indicating that intron retention may be involved in human heart development. Furthermore, the large subset of genes observed in this study underwent multiple types of AS with obviously associated changes during the foetal-to-adult transition, suggesting that the AS transition may be involved in human heart development.

In addition to its central role in increasing transcriptome complexity and proteomic diversity, AS also drives decisive physiological changes. Although a few studies have examined AS transitions during normal physiological changes, the biological functions of AS in the human foetal-to-adult transition remain unknown^7^,^10^,^27^. In this study, we used GO analysis to identify functional groups of AS events altered in foetal or adult hearts alone or simultaneously in both. Notably, foetal- and adult-specific AS events were enriched in mainly cell proliferation functions and energy-specific categories, respectively. Such splicing transitions during human heart development have also been observed in mouse and chicken heart development^13^. In addition, the splicing transitions of several genes such as calcium channel beta2 *CACNB2*, tropomyosin 1 a (*TPM1a*), muscleblind-like 1 (*MBNL1*), cardiac troponin T (*cTNT*), and disabled-1 (*Dab1*) have been demonstrated to be conserved during heart development in human, mouse, and other animals^28^,^29^,^30^,^31^,^32^,^33^. Furthermore, in consistent with the previous finding during rat heart development, α-MHC (high ATPase) and β-MHC (low ATPase) were found to be differentially expressed during human heart development^34^. The isoform β-MHC was predominantly expressed in the late foetal life^34^. Soon after birth, β-MHC shift to the isoform α-MHC, which predominantly expressed in the adult^34^. Our study further supported the evidence that a large proportion of the splicing transitions that are associated with heart development are conserved. Therefore, these conserved splicing transitions from a foetal to adult pattern strongly suggest that AS may have determinative effects in heart development.

Previous studies have revealed that differentially expressed genes (DEGs) and specific AS events could regulate genes separately or cooperatively, which has been proven to play important regulatory roles in physiological processes^13^,^17^. The RNA-sequencing analysis provided the AS and gene expression levels in foetal-to-adult transitions simultaneously, therefore, both AS and gene expression levels in foetal-to-adult transitions were analysed. In this study, we found that most of the foetal-specific AS events showed significant changes in gene expression levels. The GO analysis of the intersected foetal-specific AS and differentially expressed genes revealed that the most highly enriched GO categories were those related to cell proliferation. This observation suggests that both the AS transitions and altered gene expression levels were important regulatory components of human heart development. In contrast, the majority of genes are known to undergo AS transitions without changes in gene expression during mouse heart development^13^, indicating that there are separate regulatory mechanisms for gene expression and AS. The regulation of AS in the mouse heart development occurs through changes in isoform switching rather than total gene expression variations. The different regulatory components involved in human and mouse heart development may simply be due to species differences. Therefore, our results suggest that post-transcriptional splicing as well as transcriptional gene expression regulatory mechanisms may be involved in the switch from human foetal-to-adult gene expression processes.

Another finding of this genome-wide study was that lncRNAs undergoing AS events might not be involved in human heart development. Although data have suggested that lncRNAs play important roles in cell proliferation and developmental transitions, few studies have investigated the role of lncRNAs undergoing AS events during developmental transitions^35^,^36^,^37^. We found that AS events were more likely to occur in protein-coding genes than in lncRNA, and the AS transitions between the foetal and adult hearts occurred mainly in protein-coding genes rather than in lncRNA. These data indicate that the regulation of AS during human heart development did not occur directly through splice shifting of lncRNAs. Nonetheless, lncRNA may affect the splicing regulators of protein-coding genes, which could further regulate developmental transitions. A previous study demonstrated that lncRNA interact with nuclear AS regulators to modulate the splicing patterns during specific developmental transitions^38^. In addition, another nuclear-retained lncRNA, metastasis associated lung adenocarcinoma transcript 1 (MALAT1) has been reported to modulate AS patterns by regulating serine/arginine splicing factor phosphorylation^39^. Therefore, lncRNA is likely to regulate the human foetal-to-adult transition by modulating AS patterns in protein-coding genes. Further investigations are needed to elucidate the interactions between lncRNAs and protein-coding genes undergoing AS during human heart development, which may reveal novel regulatory mechanisms.

Collectively, the results of this study are the first to provide a profile of the AS transition between human foetal and adult hearts using high-throughput sequencing methods. We found that extensive AS transitions as well as differentially expressed genes between foetal and adult hearts may have a determinative effect during human heart development. Further examination of the specific regulatory mechanisms of AS transitions in foetal and adult hearts during development may provide feasible strategies for the restoration of proliferative competence in the adult heart.

## Methods

### RNA-sequencing dataset

The RNA-sequencing data used in this study were obtained from the European Nucleotide Archive (ENA, http://www.ebi.ac.uk/ena/) and encode project (www.encodeproject.org). The 2 × 100-bp paired-end and 36-bp single-end RNA-seq data of the foetal hearts were downloaded with two accession numbers each, SRR1998058, SRR1998059, SRR643778 and SRR643779, respectively. The 2 × 100-bp paired-end RNA-seq data of the adult hearts were downloaded with accession numbers SRR2138381, SRR2014232, ERR315356_1, and ERR315430_1. Before the data analysis, the low-quality reads were first filtered out to obtain clean reads. The low-quality reads were removed if >50% of the bases in one read were low-quality or >10% of the bases were unknown (N bases). Then, the clean reads were mapped to the human genome (hg19, http://genome.ucsc.edu) using SOAPaligner/SOAP2^40^. The resulting alignment was reconstructed using Cufflinks^41^. The RefSeq and Ensembl transcript databases were used as annotation references for the mRNA analyses while the NONCODE version 4.0 (http://www.noncode.org/NONCODERv4) was used for the lncRNA analyses^42^.

### Computing differentially expressed genes

The read counts of each transcript were normalised to the length of the individual transcript and the total mapped read counts in each sample and were expressed as reads per kilobase of exon per million mapped reads (RPKM)^43^. A transcript was defined as present when it was detected with an RPKM ≥1 in four samples within one group. The gene expression differences were evaluated using Student’s *t*-tests. The *P*-values (two-tailed) were adjusted using the Benjamini and Hochberg FDR^44^. Differentially expressed genes were defined as those with changes of at least 2.0-fold between a pair of samples at an FDR of 0.05.

### Genes and detection of AS events

The pipeline used for detecting AS events included two main steps. First, we used SOAPsplice to map the reads to the human reference sequence, and the splice junctions were reported based on the alignment of the junction reads^45^. For the SOAPsplice, we used default parameters as much as possible; the intact and splice alignment cut-offs were set at three and one mismatches, respectively. Second, based on the AS mechanisms, we used both the splice junctions, including those that were known and reported in the RefSeq and the mapping results to detect four basic AS events. These events were the alternative 5′ and alternative 3′ splice sites, intron retention, and exon skipping. A potential junction site was considered if there were more than two unambiguous reads mapped to multiple positions within the splice junction region as well as a minimum match of five bases on each side of the junction. Specific AS events were defined as only expressed within one group (foetal or adult).

### Visualisation using integrative genomics viewer (IGV)

The case studies were explored using the integrative genomics viewer (IGV, version 2.3.32) free software available from www.broadinstitute.org/igv 46. To visualise the read coverage signal map of the foetal and adult hearts in the IGV, the binary alignment/map (BAM) files were saved as an IGV-compatible format.

### Real-time PCR validation of AS events

The human cardiac myocyte-adult and -foetal cDNA (catalogue #6214 and 6204, respectively) were purchased from ScienceCell Research Laboratories (San Diego, CA, USA). In the neonatal rats, the total RNA from different tissues (the heart, liver, lung, skeletal muscle, and skin) was isolated using Trizol (Invitrogen) according to the manufacturer’s protocol. For the reverse transcription, 1 μg of total RNA was used to generate the first strand cDNA using an Oligo-dT primer.

Then, 1 μl of each cDNA product was used for the qPCR amplification using a SYBR Green PCR kit (Takara, Dalian, China) and a LightCycler 480 II (Roche Diagnostics, Basel, Switzerland) under the following conditions (40 cycles): 95 °C denaturation for 30s, 40 cycles at 95 °C for 5 s, 60 °C for 1 min, cooling at 50 °C for 30 s. At the end of the qPCR runs, a melting curve was created. The primers were designed and verified using the primer 3, Oligo 7.0, and MFEprimer-2.0^47^. The primers used in this study are shown in Table 2, and each sample was tested in triplicate. The mRNA level was normalised to glyceraldehyde 3-phosphate dehydrogenase (GAPDH), and the relative mRNA expression was calculated using the 2^−ΔΔCt^ method. The PCR products were also separated on 1.5% agarose gels.

### Hierarchical clustering analyses

We performed a cluster analysis of the gene expression patterns using the Cluster 3.0 and JavaTree view software^48^,^49^. The gene expression differences were clustered using the hierarchical complete linkage clustering method using the Euclidean distance and average linkage.

It is noteworthy that we added two additional steps prior to clustering the genes. First, genes with only one read covered were removed for lack of reliability. Then, we pre-treated the dataset using Microsoft Excel. All data values (x) were replaced with their log2(x) values, and the column-wise means were subtracted from the values in each data column such that the mean value of each column was 0. Finally, the red- and green-coloured entries correspond to fully induced and repressed expressions, respectively.

### Functional analyses

The genes lists were submitted online to the database for annotation, visualization and integrated discovery (DAVID) for GO enrichment and KEGG pathway analysis^50^. A hypergeometric test was performed using the Benjamini and Hochberg FDR with default parameters to adjust the *P*-value. The enrichment was considered significant when the *P*-value < 0.05.

### Statistical and computational methods

For the gene expression analysis, the statistical significance was assessed using the Student’s *t*-test as described above. For the GO enrichment and KEGG pathways analysis, the Benjamini and Hochberg FDR was performed as described above. All other computational procedures were carried out using in-house programs written in R or analysed in Microsoft Excel and Stata 12.0. A two-sided *P*-value < 0.05 was considered statistically significant for all analyses.

### Availability of supporting data

The data sets supporting the results of this article are included in the article and the Additional files.

## Additional Information

**How to cite this article**: Wang, H. *et al*. Genome-wide analysis of alternative splicing during human heart development. *Sci. Rep.* **6**, 35520; doi: 10.1038/srep35520 (2016).

## Supplementary Material

Supplementary Information

## Figures and Tables

**Table 1:** Summary of RNA-seq results of reads.

	Total reads	Mapped Reads (%)	Unique match (%)
Foetal 1	74678048	93.0	82.4
Foetal 2	52457369	91.2	81.0
Foetal 3	169435810	85.55	77.23
Foetal 4	131730114	86.16	81.21
Adult 1	31449650	89.7	83.9
Adult 2	30835782	89.7	83.8
Adult 3	96915682	65.05	61.28
Adult 4	135287130	87.73	69.22

**Table 2:** Primer sequences.

Gene	Primer	Primer sequences(5′-3′)
PUM1-isoform1	Forward	TTCCTTCAGACCAGCAGGTAAT
	Reverse	TGCCCGTCTCAGACTCTACA
PUM1- isoform 2	Forward	ACCCCCATTGGACACAGTTT
	Reverse	CTCATTAATTACCTGCTGGTCTG
TNNT2- isoform 1	Forward	CTGCTGTTCTGAGGGAGAGC
	Reverse	CACCAAGTTGGGCATGAACG
TNNT2- isoform 2	Forward	AGAGGAGGAGGAGCTCGTTT
	Reverse	CCCACTTTTCCGCTCTGTCT
ANAPC11-isoform 1	Forward	GACGAGCTGCGGAGACG
	Reverse	TGCAATGCATGTGGAAGCAG
ANAPC11- isoform 2	Forward	CTGCAGGATGGCATTTAACGG
	Reverse	GGGTCTGGGGACCTAGAAGAC
GAPDH	Forward	AGAAGGCTGGGGCTCATTTG
	Reverse	AGGGGCCATCCACAGTCTTC
