# Supplementary material for: Genome-wide analysis of alternative splicing during human heart development
Source: Sci Rep. 2016 Oct 18;6:35520. doi: 10.1038/srep35520 (PMC5067579; doi:10.1038/srep35520)
Supplement: Supplementary Information [file srep35520-s1.pdf]

Genome-wide analysis of alternative splicing during human heart development

He Wang, MD[1,3*]; Yanmei Chen, MD[1*]; Xinzhong Li, MD[1]; Guojun Chen, MD[1]; Lintao Zhong, MD[1]; Gangbing Chen MD[1],Yulin Liao, MD[1]; Wangjun Liao, MD, PhD [2]; and Jianping Bin, MD, PhD[1,†]

[1]Department of Cardiology, State Key Laboratory of Organ Failure Research, Nanfang Hospital, Southern Medical University, Guangzhou510515, China

[2]Department of Oncology, Nanfang Hospital, Southern Medical University, Guangzhou 510515, China

[3]Department of Cardiology, Second Affiliated Hospital of Nanchang University, Jiangxi 330006, China.

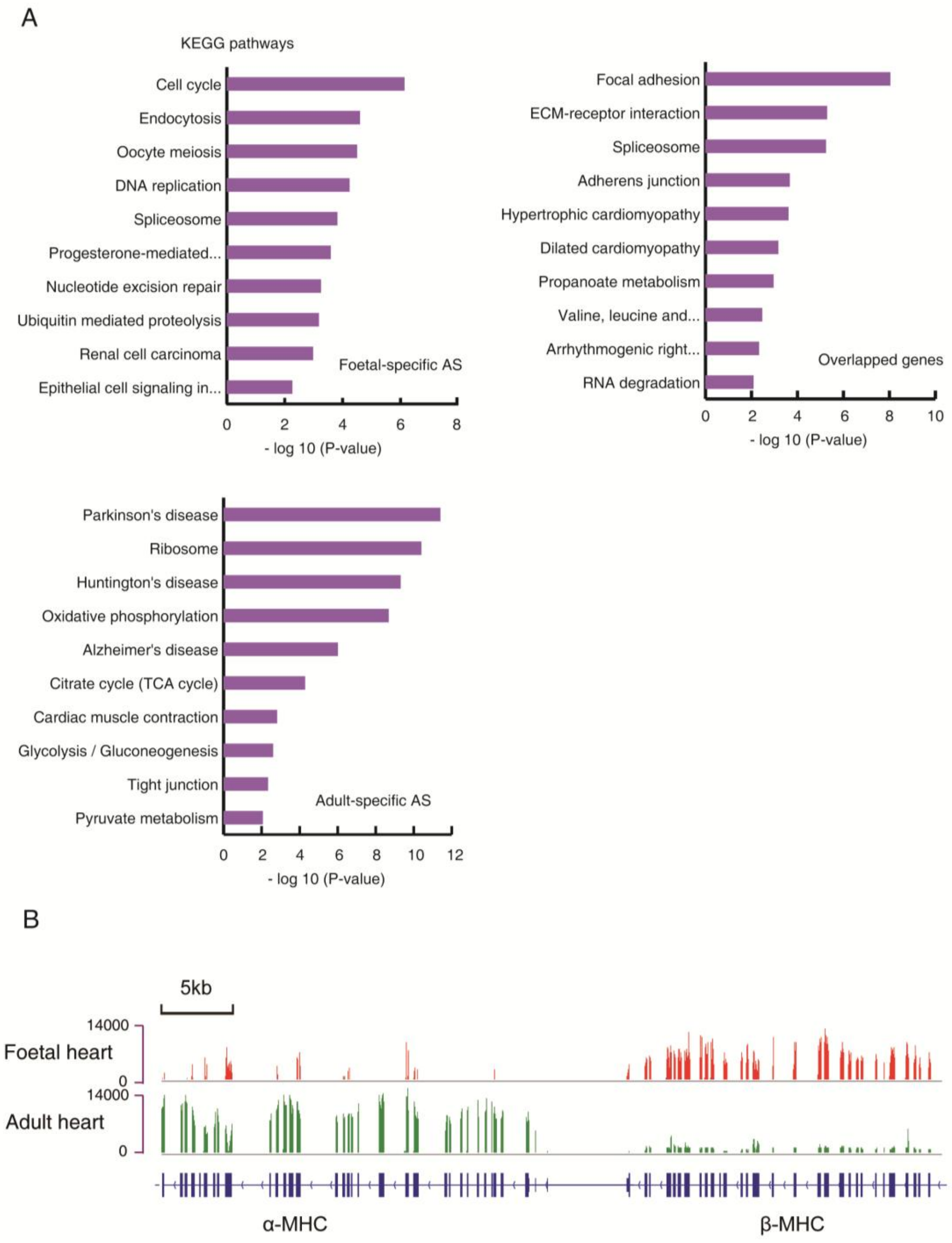

Figure S1 The Kyoto Encyclopaedia of Genes and Genomes(KEGG) pathway analysis of specific AS genes. (A) KEGG pathway analysis of foetal/adult-specific AS genes. (B) RNA-sequencing read density plot for α-MHC and β-MHC between fetal and adult hearts. Foetal-specific AS, gene with specific AS events in foetal hearts; overlapped genes, overlapped genes of foetal- and adult-specific AS; adult specific AS, gene with specific AS events in adult hearts.

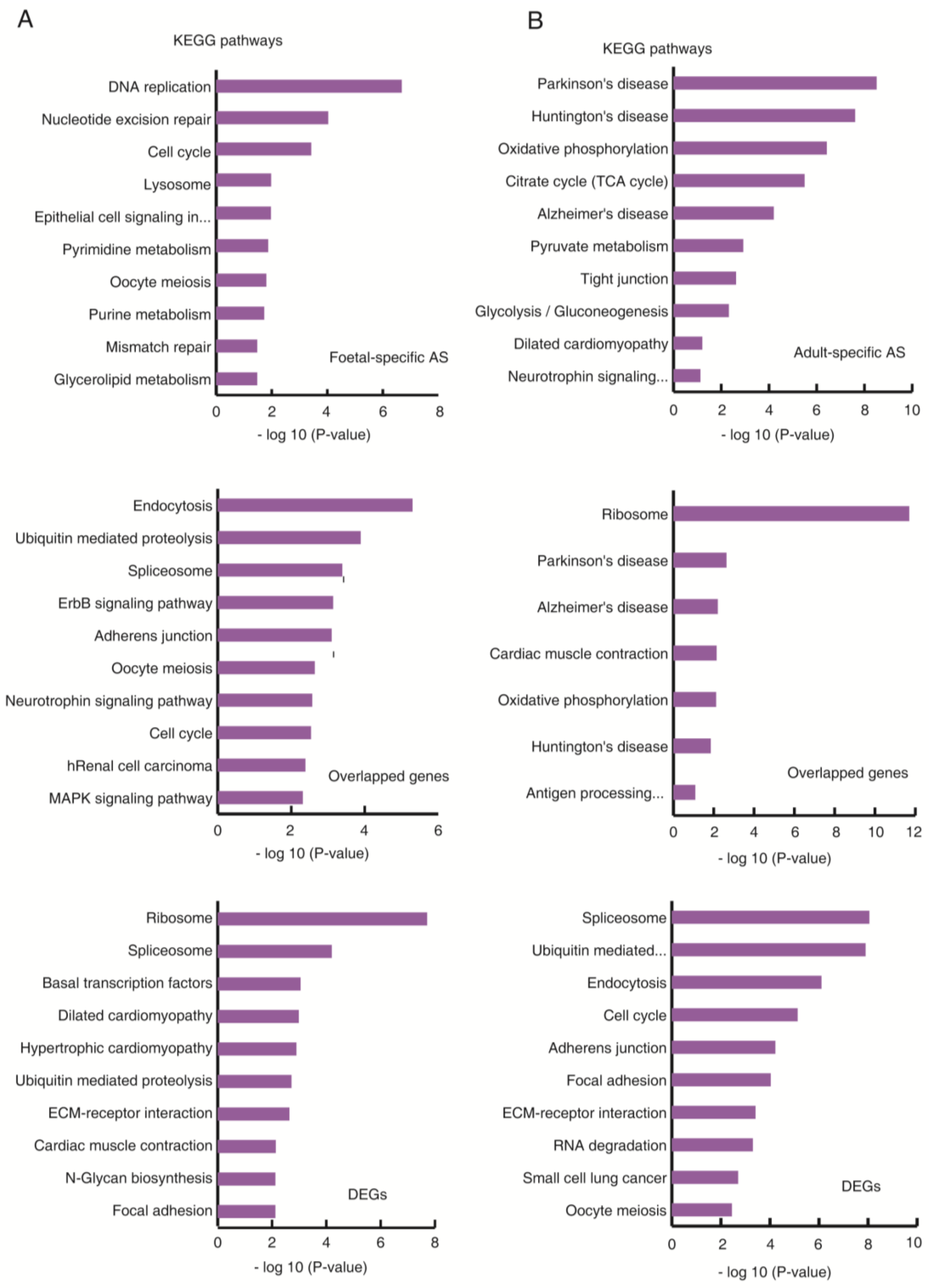

Figure S2. The KEGG pathway analysis of specific AS genes and DEGs. (A)The KEGG pathway analysis of foetal specific AS and DE genes. (B) The KEGG pathway analysis of adult specific AS and DE genes. DEGs, differentially expressed genes between foetal and adult hearts; foetal specific AS, gene with specific AS events in foetal hearts; adult specific AS, gene with adult AS events in adult hearts; overlapped genes, overlapped genes of foetal(A) or adult(B) specific AS and DEGs.

Table S1. Foetal specific AS genes differential expressed associate with cell cycle

Gene ID	Chr	Strand	Alternative splite (h19)
472	chr11	+	108142134-108143257, 108203628-108204611, 108205837-108206570
545	chr3	-	142259877-142261505
580	chr2	-	215632379-215633954
699	chr2	-	111398783-111398882, 111399042-111399217, 111399381-111399694, 111411101-111413314, 111413494-111414611, 111415223-111415985, 111416320-111417553, 111425283-111425372
815	chr5	-	149607850-149610866
817	chr4	-	114430793-114430828, 114375672-114376880, 114378720-114381301, 114434527-114434984, 114424091-114424133, 114429391-114429424
904	chr12	-	49091967-49093559
905	chr2	+	135700245-135703669, 135710282-135710798
983	chr10	+	62547817-62547988
991	chr1	+	43825493-43825638
994	chr20	+	3780977-3781107, 3781146-3781388, 3781513-3781616, 3782748-3782926, 3783024-3783555
1020	chr7	-	150753724-150753821
1062	chr4	-	104068804-104069999, 104070566-104072374, 104079616-104079769, 104082615-10408429, 104096061-104097115, 104117196-104117318
1063	chr1	+	214825230-214826169
1762	chr19	-	46287549-46287897, 46287974-46288850
2033	chr22	+	41533795-41536142, 41542821-41543839, 41543951-41545040, 41545180-41545763, 41546203-41547835, 41558784-41560055
2873	chr17	+	80012385-80012686, 80014643-80014714, 80014851-80014927, 80014715-80014850
3054	chrX	-	153218410-153219056, 153222512-153222763, 153224218-153224780, 153225613-153225684 153227100-153227671, 153227758-153228674, 153228885-153229573, 153229736-153230027
3619	chr11	+	61906478-61907704, 61912762-61913099
3838	chr17	+	66042038-66042618
3925	chr1	-	26231230-26232878
3980	chr17	+	33325316-33325621, 33326469-33326799
4288	chr10	-	129914272-129914753
4297	chr11	+	118370136-118370548, 118370629-118371700

4751	chr1	+	211842675-211843621, 211843750-211844542
4853	chr1	-	120491190-120491628, 120462237-120462850, 120468434-120469120
5310	chr10	-	75206249-75206334, 75204581-75206248
5701	chr7	+	102996140-102996240
5716	chrX	-	107331083-107331181
5810	chr5	-	34909462-34911657
5932	chr18	+	20573603-20573682, 20596790-20596887
6045	chr1	+	185062192-185062408
6598	chr22	+	24133942-24134054
6787	chr3	-	52780921-52783706
6790	chr20	-	54967223-54967351, 54945397-54945539 54958233-54959324, 54965611-54965721
6868	chr2	-	9676052-9676825
6929	chr19	-	1612205-1612429, 1621970-1622051
6934	chr10	+	114910741-114910882
7283	chr17	+	40764525-40764963, 40766431-40766512
7465	chr11	+	9597841-9598032, 9607075-9607994
8239	chrX	+	41088819-41089080, 41055613-41055843 41055992-41056615
8438	chr1	+	46743653-46743742
8450	chrX	-	119672065-119672513, 119680472-119680989
8453	chr10	-	35318569-35320226, 35320315-35320417
8658	chr8	+	9592595-9592885, 9623308-9623747
8697	chr5	-	137542235-137542369, 137527225-137527549 137527627-137527956
9055	chr15	-	91512753-91512853
9126	chr10	+	112343307-112343597, 112349750-112350168 112350331-112350747,112359571-112360195 112360305-112360778, 112362424-112362581 112362761-112362940
9181	chr1	-	155934778-155934919
9493	chr15	+	69717620-69718521, 69718522-69718673 69732417-69732645, 69737381-69738346
9611	chr17	-	16029395-16029523
9738	chr16	+	19556221-19556414
9787	chr14	-	55615192-55615310, 55647497-55647929
9793	chr11	-	46774261-46774858, 46780594-46780817 46791649-46792336, 46799103-46799687, 46799864-46800008, 46801870-46801976, 46818491-46819353, 46819520-46819631
9874	chr2	-	171850467-171853161
9918	chr12	+	6619977-6620274, 6626192-6626529 6626666-6626760, 6626850-6626943

			6635782-6636055, 6636230-6636941
			6637055-6637132, 6637258-6637337
			6638023-6638109, 6639984-6640085
			6640243-6640491
9988	chr7	+	86810658-86811542
10015	chr3	+	33870329-33870461
10179	chr11	+	114276427-114276521
10198	chr12	-	123686867-123687169, 123687632-123687795
10274	chr3	-	136140006-136141250, 136141700-136141792
10615	chr17	-	26913017-26913106
10801	chr17	+	75486942-75488701
11004	chr1	+	45219506-45220420, 45221656-45221745
11044	chr5	+	6738843-6739843
11064	chr9	+	123911140-123912446, 123914972-123916997
11104	chr6	-	149918778-149919358
11113	chr12	-	120139558-120139653, 120151100-120151271
11190	chr20	+	34054898-34055127
11258	chr9	-	34617969-34618671
22974	chr20	+	30363792-30365288, 30365442-30366614
23047	chr13	+	33332358-33332670, 33344699-33344790
23310	chr11	-	134026991-134027821, 134073094-134073547
23332	chr2	-	122144805-122145337
23435	chr1	+	11076900-11077064
23637	chr9	+	125836708-125838522, 125861829-125863843
24149	chr6	-	43309950-43310412
25836	chr5	+	37000991-37001089, 37006691-37007423
			37007577-37008108
25936	chr1	-	212955769-212957703
26586	chr13	+	53036703-53039428
27436	chr2	+	42522400-42522519
29127	chr12	-	50386161-50386331
29945	chr4	+	25404649-25407193
51529	chr17	+	79849598-79849717, 79849710-79851426
			79849718-79851426
54443	chr7	+	36463553-36464152, 36464203-36464293
			36456688-36456799
54790	chr4	+	106180927-106182914
54930	chr14	-	23420763-23420898
55183	chr2	+	152300224-152301850, 152316634-152317650
			152317804-152318754, 152318837-152318924
			152325271-152326223
55213	chr13	-	50140905-50141288
55544	chr20	+	55968334-55968389
55749	chr10	+	70515294-70516028, 70500385-70500418

56647	chr10	+	127522516-127524671
60561	chr7	+	105205905-105205975
79858	chr3	+	130887782-130889615
84962	chr14	-	23443325-23444046, 23445728-23445852
115426	chr9	+	6481767-6481990
116138	chr6	+	42986295-42986369, 42986904-42987024<br>42987105-42988403,42985256-42985433
116840	chr17	+	7852399-7852475, 7836668-7837454<br>7840136-7840480, 7851646-7851803<br>7851938-7852401, 7852476-7852701
151011	chr2	-	110332157-110332344
283431	chr12	+	100994329-100995414
348995	chr6	-	150057759-150059777, 150067199-150067510
